# DHEA administration and exercise training improves insulin resistance in obese rats

**DOI:** 10.1186/1743-7075-9-47

**Published:** 2012-05-30

**Authors:** Koji Sato, Motoyuki Iemitsu, Katsuji Aizawa, Noboru Mesaki, Ryuichi Ajisaka, Satoshi Fujita

**Affiliations:** 1 Faculty of Sport and Health Science, Ritsumeikan University, Kusatsu, Shiga, Japan; 2 Department of literature, Senshu University, Kawasaki, Kanagawa, Japan; 3 University of Tsukuba, Tsukuba, Ibaraki, Japan; 4 Department of Comprehensive Human Sciences, University of Tsukuba, Tsukuba, Ibaraki, Japan

**Keywords:** Exercise training, Insulin sensitivity, Sex steroid hormone, Obesity

## Abstract

**Background:**

Dehydroepiandrosterone (DHEA) is precursor of sex steroid hormone. We demonstrated that acute DHEA injection to type 1 diabetes model rats induced improvement of hyperglycemia. However, the effect of the combination of DHEA administration and exercise training on insulin resistance is still unclear. This study was undertaken to determine whether 6-weeks of DHEA administration and/or exercise training improve insulin resistance in obese male rats.

**Methods:**

After 14 weeks of a high-sucrose diet, obese male Wistar rats were assigned randomly to one of four groups: control, DHEA administration, exercise training, and a combination of DHEA administration and exercise training (*n* = 10 each group).

**Results:**

After 6-weeks of DHEA administration and/or exercise training, rats in the combination group weighed significantly less and had lower serum insulin levels than rats in the other groups. Moreover, the rats treated with DHEA alone or DHEA and exercise had significantly lower fasting glucose levels (combination, 84 ± 6.5 mg/dL; DHEA, 102 ± 9.5 mg/dL; control, 148 ± 10.5 mg/dL). In addition, insulin sensitivity check index showed significant improvements in the combination group (combination, 0.347 ± 0.11; exercise, 0.337 ± 0.16%; DHEA, 0.331 ± 0.14; control, 0.308 ± 0.12). Muscular DHEA and 5α-dihydrotestosterone (DHT) concentrations were significantly higher in the combination group, and closely correlated with the quantitative insulin-sensitivity check index (DHEA: *r* = 0.71, *p* < 0.01; DHT: *r* = 0.69, *p* < 0.01).

**Conclusion:**

These results showed that a combination of DHEA administration and exercise training effectively improved fasting blood glucose and insulin levels, and insulin sensitivity, which may reflect increased muscular DHEA and DHT concentrations.

## Introduction

Obesity is one of the risk factors for type 2 diabetes, cardiovascular diseases, hypertension, and dyslipidemia. The obesity and type 2 diabetes patients show lower concentrations of dehydroepiandrosterone (DHEA) and other sex steroid hormone [1-3]. Decreased DHEA levels in obese patients or those with type 2 diabetes are related to insulin-induced inhibition of enzyme activity for adrenal androgen synthesis [4]. However, DHEA administration and exercise leads to increases in muscular steroidogenesis-related enzymes and concomitant increases in plasma and muscular DHEA and 5α-dihydrotestosterone (DHT) levels in rats [5-7]. Moreover, several reports demonstrated that short-term (2 weeks) DHEA administration induced an acute decrease in blood glucose levels in mice [8,9], reduced serum insulin levels in older rats [2,10,11], and enhanced the activities of enzyme related to hepatic glucose metabolismin diet-induced or Zucker obese rats [12,13]. Recently, we have reported that acute DHEA injections improved hyperglycemia in streptozotocin (STZ)-induced diabetic rats [7]. In addition, Han and colleagues [14] demonstrated an enhanced insulin sensitivity in response to 2 weeks of wheel-running exercise and DHEA administration in 25-month-old rats, although additive effects of the treatments were not apparent.

DHEA and its sulfate derivate (DHEA-S) are precursors of sex steroid hormones, that circulate in blood before they are used by target tissues. Generally, DHEA is produced by adrenal, but several studies demonstrated that brain, liver, bone and any other tissues can also produce DHEA in both human and rodents [15-17]. Recently, we demonstrated that skeletal muscle can locally synthesize DHT from DHEA and testosterone [18]. According to our previous studies, acute aerobic exercise enhances the local bioactive androgen metabolism, and increased DHEA and DHT levels in skeletal muscle (5, 6). It is still unclear whether the combination of chronic DHEA administration and aerobic exercise training increases muscular level of DHEA and DHT, and it induces the improvement of insulin resistance more effectively in obesity.

We hypothesized that, compared with either DHEA administration or exercise training treatment alone, 6-weeks of DHEA administration combined with exercise training in obese rats would produce larger effects on steroidogenesis from DHEA to DHT in skeletal muscle and improvements in insulin sensitivity. To test this hypothesis, we investigated fasting blood glucose, insulin levels, the quantitative insulin-sensitivity check index (QUICKI) as an index of insulin sensitivity after 6-weeks of DHEA administration, exercise training, or both in rats with diet-induced obesity.

## Methods

Ethical approval for this study was obtained from the Committee on Animal Care at the University of Tsukuba. Male Wistar rats (220–250 g, 10 weeks old; Charles River Japan, Kanagawa, Japan) were cared for according to *The Guiding Principles for the Care and Use of Animals* based on the Declaration of Helsinki. The Wistar rats were housed individually in an animal facility under controlled conditions (12:12-h light–dark cycle). The rats were allowed water *ad libitum* and placed on a purified high-sucrose diet (68% of kcal from sucrose, 20% from protein, and 12% from fat) for 14 weeks, according to a previous study with minor modifications [19]. After 14 weeks of the high-sucrose diet, the animals were randomly assigned to one of four groups: sedentary rats administered sesame oil (control group, *n* = 10), obese rats treated with DHEA (DHEA group, *n* = 10), obese rats subjected to exercise training (exercise group, *n* = 10), and obese rats treated with DHEA and subjected to exercise training (combination group, *n* = 10). DHEA was obtained from Wako Pure Chemical Industries (Osaka, Japan). All animals continued on the high-sucrose diet during the 6-week experimental period. In the DHEA and combination groups, DHEA (1 mg/kg body weight) dissolved in sesame oil was administered orally every day for 6 weeks. In the control group, the same amount of vehicle (sesame oil) was administered orally every day. Body weight and dietary intake were measured every week during the experiment. Post-training experiments in trained rats were performed 48 h after the last round of exercise training. After all other measurements were obtained the soleus and gastrocnemius muscles were quickly removed, weighed, rinsed in ice-cold saline, and frozen in liquid nitrogen.

### Exercise protocol

The obese exercise training group was trained on a rodent treadmill (KN-73, Natsume Seisakusyo, Tokyo, Japan) at about 10–25 m/min over a period of 3 days for accustomed to the treadmill. The rats then ran on the treadmill for 1 h at 25 m/min without incline 5 days/week for 6 weeks. The intensity, duration and time of the exercise was kept constant during the rest of the training period.

### Protein assay and muscle concentrations of DHEA and DHT

Muscle specimens were homogenized in 20 mM Tris–HCl (pH 7.8), 300 mM NaCl, 2 mM ethylenediaminetetraacetic acid (EDTA), 2 mM dithiothreitol (DTT), 2% nonident P-40, 0.2% sodium lauryl sulphate (SDS), 0.2% sodium deoxycholate, 0.5 mM phenylmethylsulfonyl fluoride, 60μg/ml aprotinin, and 1 μg/ml leupeptin. Homogenates were rotated slowly for 30 min at 4°C and then centrifuged at 12,000 × *g* for 15 min at 4°C. The protein concentration of the resulting supernatant was determined. For the determination of DHEA and DHT levels, muscle samples were diluted by 200 times with each assay buffer. The levels of DHEA and DHT in skeletal muscle extracts were determined using Enzyme-Linked Immuno Sorbent Assay (ELISA) kit (Assay Designs, Ann Arbor, MI, IBL Hamburg, Germany). The techniques and materials used in these analyses followed the manufacturer’s protocol. The immobilized polyclonal antibodies were raised against DHEA and DHT, whereas the secondary horseradish-peroxidase-coupled antibodies were monoclonal. Optical density at 450 nm was measured on a microplate reader (BioLumin 960; Molecular Dynamics, Tokyo, Japan). All samples were assayed in duplicate.

### Glucose and insulin concentrations

Serum insulin concentrations were measured using an ELISA kit (Shibayagi Co, Gunma, Japan), according to the manufacturer’s protocol. All samples were assayed in duplicate. Optical density at 450 nm was qualified using a microplate reader (BioLumin 960; Molecular Dynamics, Tokyo, Japan). Fasting glucose was assessed from the tail vein before and after the treatment period under overnight fasting condition. Glucose concentrations were assessed three times from the tail vein using a blood glucose meter (Ascensia, Bayer HealthCare, Tokyo, Japan).

### Insulin sensitivity

QUICKI was calculated according to the previous study from fasting glucose and insulin vaues [20-22]. QUICKI = 1/[log (I_0_) + log (G_0_)], where I_0_ is fasting insulin (μU/ml), and G_0_ is fasting glucose (mg/dl).

### Statistical analysis

All values are expressed as means ± SE. Statistical evaluations were performed using repeated measures two-way ANOVA (time × group) for weight changes. A post-hoc comparison test was used to correct for multiple comparisons (Bonferroni test) when analyses revealed significant differences. For ANOVA, *P* < 0.01 was considered significant. Relationships between sex steroid hormone concentrations and the quantitative insulin-sensitivity check index (QUICKI) was determined using Pearson correlation coefficients.

## Results

### Body weight

**Figure 1:**
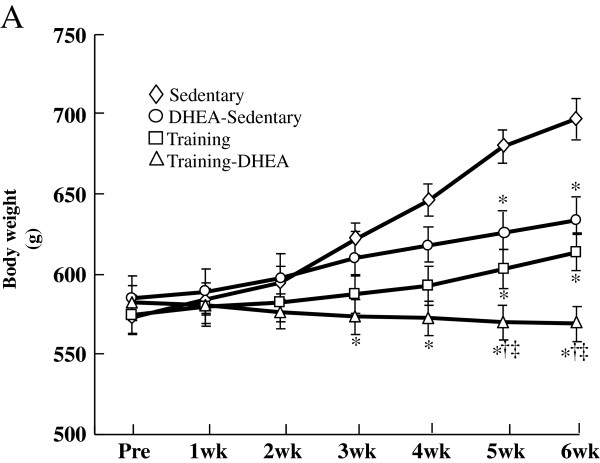
** Body weight before and after dehydroepiandrosterone (DHEA) treatment and exercise training in obese rats fed a high-sucrose diet.** Diamonds: Control group. Circles:DHEA treatment group. Squares: Exercise training group. Triangles: Combination group Data are means ± SE from 10 animals. Pre: before DHEA treatment and/or exercise training. The x axis represents the weeks of DHEA treatment and/or exercise training. * *p* < 0.01 compared with the control group. †*p* < 0.01 compared with the DHEA group. ‡ *p* < 0.01 compared with the exercise training group.

Body weight before the treatment periods did not differ among the groups. After 6 weeks of DHEA administration and/or exercise training, body weight was significantly (*p* < 0.01) lower in the combination group than in the control group. After 5 weeks, body weight was significantly (*p* < 0.01) lower in the combination group than in the DHEA group or the exercise group (Figure 1). Final body weights in the DHEA and exercise groups were significantly lower than in the control group. Moreover, the combination group showed a significantly lower body weight than the other groups (Figure 1).

### Dietary intake, muscle weight and epididymal fat weight

Exercise training and DHEA administration did not decrease food intake of the rats. Average food intake during the 6 weeks was 19.74 ± 0.62 g/day in the control group, 19.64 ± 0.37 g/day in the DHEA group, 19.61 ± 0.43 g/day in the exercise group, and 19.59 ± 0.53 g/day in the combination group. No significant difference in the food intake was found among groups during the treatment period (Table 1).

**Table 1:** Animal characteristics

	**Sedentary (n = 10)**	**DHEA (n = 10)**	**Training (n = 10)**	**Training-DHEA (n = 10)**
Muscle weight (g/body weight)				
Soleus	0.32 ± 0.02	0.48 ± 0.04 *	0.56 ± 0.03 *†	0.57 ± 0.04 *†
Gastrocnemius	3.63 ± 0.6	4.31 ± 0.3 *	4.66 ± 0.3 *†	4.73 ± 0.3 *†
Epididymal fat (g)	38.2 ± 4.8	27.4 ± 3.6 *	33.8 ± 2.8	23.7 ± 4.1 *‡
Dietary intake(g/day)	19.74 ± 0.62	19.64 ± 0.37	19.61 ± 0.43	19.59 ± 0.53
Pre-fasting glucose(mg/dl)	154 ± 11.5	159 ± 10.2	162 ± 8.7	158 ± 9.8
Post-fasting glucose(mg/dl)	148 ± 10.5	102 ± 9.5 *	92 ± 7.8 *	84 ± 6.5 *†

**DHEA:**dehydroepiandrosterone.

**Pre-fasting glucose:** fasting glucose level before DHEA administration and/or exercise training.

**Post-fasting glucose:** fasting glucose level after DHEA administration and/or exercise training.

Data are means ± SE.

* *P* < 0,05, vs Sedentary control group.

†*P* < 0.05, vs DHEA treatment group.

‡*P* < 0.05, vs Training group.

Table 1 shows the weights of the soleus and gastrocnemius muscles after the treatment period. Marked increases in the weight of the soleus and gastrocnemius muscles were observed in the exercise and combination groups compared with the control and DHEA groups. The soleus and gastrocnemius muscles weighed significantly more in the DHEA group than in the control group (*p* < 0.01, Table 1). Epididymal fat weight was significantly decreased in combination group compared to control and exercise group (*p* < 0.01, Table 1).

### Fasting glucose levels, serum insulin levels, and QUICKI

The fasting glucose levels were not significantly different among groups before the treatment periods (Table 1). After 6 weeks of DHEA administration and/or exercise training, fasting glucose levels were significantly lower in the DHEA, exercise, and combination groups than in the control group. Moreover, fasting glucose levels in the combination group were significantly lower than in the DHEA group (Table 1).

**Figure 2:**
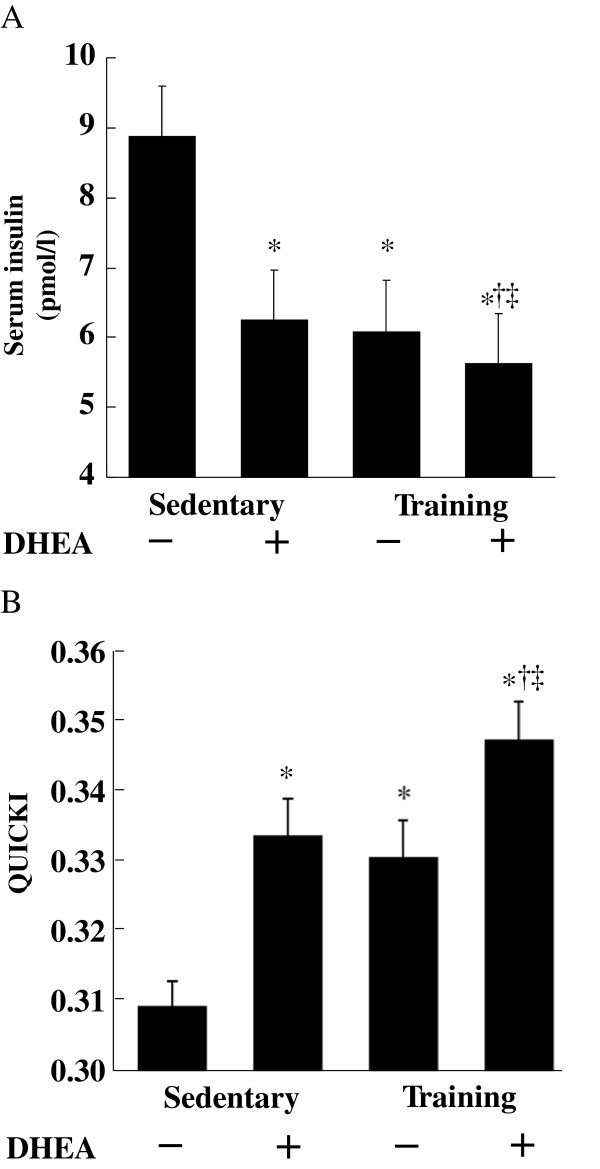
** Effects of DHEA treatment and/or exercise training on serum insulin levels (A) and the QUICKI (B).** Data are the means ± SE from 10 animals. * *p* < 0.01 compared with the control group. †*p* < 0.01 compared with the DHEA group. ‡ *p* < 0.01 compared with the exercise training group.

After 6 weeks of DHEA administration and/or exercise training, the serum insulin levels were significantly lower in the DHEA, exercise, and combination groups than in the control group. Serum insulin levels were also significantly lower in the combination group than in the other groups (Figure 2A). Similarly, QUICKI were significantly higher in the DHEA, exercise, and the combination groups than in the control group (*p* < 0.01. Figure 2B). Of particular interest, the QUICKI were significantly higher in the combination group than in the other groups (*p* < 0.01). No significant correlation between body weight and fasting glucose, or body weight and QUICKI was found in the present study.

### Intramuscular DHEA and DHT concentrations

**Figure 3:**
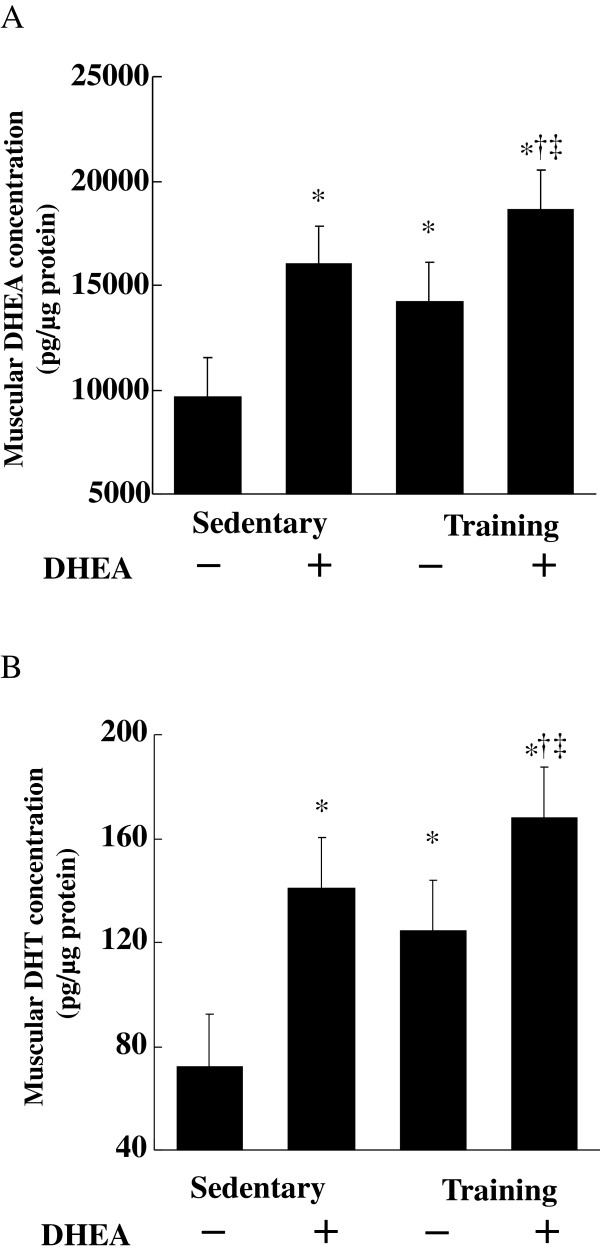
** Effects of DHEA treatment and/or exercise training on muscle DHEA and 5α-dihydrotestosterone (DHT) concentrations.** Data are means ± SE from 10 animals. *p < 0.01 compared with the control group. †*p* < 0.01 compared with the DHEA group. ‡ *p* < 0.01 compared with the exercise training group.

**Figure 4:**
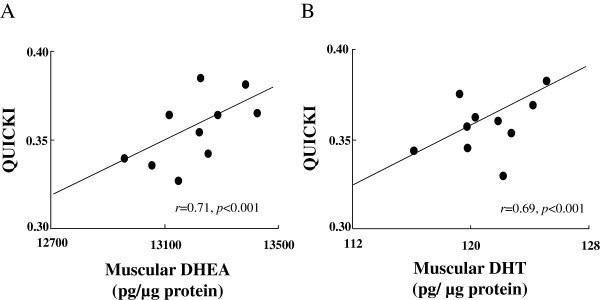
Correlations between muscular DHEA and the QUICKI (A) and muscular DHT and the QUICKI (B) in combination treatment groups.

Intramuscular DHEA and DHT concentrations were significantly greater in the DHEA, exercise, and combination groups than in the control group. The DHEA and DHT concentrations were also significantly greater in the combination group than in either the DHEA or exercise group (Figures 3A and 3B). Of note, intramuscular DHEA and DHT concentrations significantly correlated with QUICKI (DHEA: *r* = 0.71, *p* < 0.001; DHT: *r* = 0.69, *p* < 0.001. Figure 4).

## Discussion

Results from this study demonstrated that 6-weeks of DHEA treatment with exercise training induced larger decreases in fasting glucose levels than results observed for DHEA administration alone. The combined interventions were more beneficial for insulin resistance and body weight than either DHEA administration or exercise training alone. These effects in the combination group may have reflected significant increases in muscular DHEA and DHT levels, which closely correlated with QUICKI.

In the present study, both exercise training and DHEA administration significantly increased muscular concentrations of DHEA and DHT, and these increases were even more pronounced when these treatment modalities were combined. In fact, muscular DHEA and DHT concentrations significantly correlated with QUICKI in this study. Recently, a single DHEA injection in rats with STZ-induced diabetes was shown to reverse impaired GLUT-4-related signaling in muscle, such as Akt/PKCζ/λ activity and GLUT-4 translocation [7]. Additionally, short-term exercise training is known to improve blood glucose levels and accelerate muscular glucose uptake and utilization [23]. Therefore, the combination of DHEA administration and exercise training may additively or synergistically improve blood glucose levels and activate the glucose uptake in skeletal muscle. According to Han et al. [14], plasma insulin levels decreased and the glucose infusion rate was increased by DHEA treatment and voluntary running-wheel training in 25-month-old rats. Additive effects were not observed in the combination group, however. In the present study, a 6-week regimen of DHEA administration and exercise training resulted in larger improvements in hyperglycemia and insulin resistance in the obese rats. Consequently, a longer treatment period, such as the 6-week regimen used in this study, may be required to obtain additive benefits for improvement of insulin sensitivity in diet-induced obese rats.

Although DHEA administration and exercise training each produced beneficial effects, 6-weeks of combination treatment were more effective for obesity. The precise mechanisms that reduced abdominal fat weight in the combination group remain unclear, yet we can propose several plausible hypotheses. 2 weeks of DHEA administration has been shown to activate fatty acid metabolism-related enzymes, such as long-chain fatty acyl-coenzyme A synthase, and to increase free CoA levels in liver [12,13]. In addition, exercise training is known to reduce adipogenesis via upregulation of fatty acid metabolism and increased energy expenditure [24]. Therefore, 6-weeks of combination treatment may have promoted additive reductions in abdominal fat volume.

Patients with metabolic syndrome have lower DHEA and DHEA-S levels [25]. We recently demonstrated that DHEA enhanced GLUT-4-relatedsignaling in cultured skeletal muscle cells [18]. Furthermore, rats with STZ-induced diabetes had lower muscle DHEA concentrations, and acute DHEA injections improved GLUT-4-relatedsignaling with increased muscular DHEA and DHT levels. Therefore, muscular DHEA and DHT levels appear to be related to blood glucose levels in obesity and type 2 diabetes. The present study showed that DHEA administration, exercise training, and combination of the two modalities significantly increased basal muscular DHEA and DHT levels. Moreover, brief exercise significantly increases muscular levels of DHEA and steroidogenesis-related enzymes [5,6]. Exercise training is beneficial for patients with insulin resistance and hyperinsulinemia [26,27]. Although exercise training has been shown to improve insulin resistance and glucose metabolism in obese patients with type 2 diabetes, most effects are observed after longer exercise programs. Therefore, combination treatment may be more beneficial than either therapy alone. Increasing basal muscular DHEA and DHT levels with DHEA and regular exercise may provide additive benefits for reducing abdominal fat weight and improving insulin resistance in obesity. However, the present study was conducted with relatively small number of experiments; therefore, further studies are warranted to confirm the current findings and to clarify the molecular mechanisms for combined benefits of DHEA and exercise training.

Although this combined treatment modalities may provide a new therapeutic avenue for obesity and insulin resistance related to obesity, previous studies have reported some mixed results. While one study indicated that DHEA administration improved insulin sensitivity in elderly people [28,29], others demonstrated no improvement in insulin sensitivity or body composition [30]. Therefore, further studies are necessary to clarify the effect of DHEA supplementation not only in healthy adults but also in obese or diabetic patients.

In conclusion, the results from this study demonstrated that 6-weeks of DHEA administration combined with exercise training produced larger benefits for insulin sensitivity, body weight, and abdominal fat compared with DHEA administration or exercise training alone.

## Abbreviations

DHEA, Dehydroepiandrosterone; DHEA-S, DHEA sulfate; DHT, 5α-dihydrotestosterone; QUICKI, Quantitative insulin-sensitivity check index.

## Competing interests

The authors declare that they have no competing interests.

## Authors’ contribution

SK: AB, IM: ES, AK: ES, MN: ES, AR: FG, FS: FG. All authors read and approved the final manuscript.
